# Tracing motor neurons and primary sensory afferents of the monkey spinal cord with cholera toxin subunit B

**DOI:** 10.4103/NRR.NRR-D-24-00995

**Published:** 2025-03-25

**Authors:** Ziyu He, Zhixian Liu, Wenjie Xu, Ruoying Zhang, Shu Fan, Wei Wang, Xiaolong Zheng

**Affiliations:** 1 Department of Neurology, Tongji Hospital, Tongji Medical College, Huazhong University of Science and Technology, Wuhan, Hubei Province, China; 2 Hubei Key Laboratory of Neural Injury and Functional Reconstruction, Huazhong University of Science and Technology, Wuhan, Hubei Province, China; 3 Key Laboratory of Neurological Diseases of Chinese Ministry of Education, School of Basic Medicine, Tongji Medical College, Huazhong University of Science and Technology, Wuhan, Hubei Province, China

**Keywords:** cholera toxin subunit B, interneuron, Macaca Mulatta, monkey, motor neuron, neuron tracing, primary sensory afferents, rhesus macaque, sciatic nerve, spinal cord

## Abstract

Nonhuman primates are increasingly being used as animal models in neuroscience research. However, efficient neuronal tracing techniques for labeling motor neurons and primary sensory afferents in the monkey spinal cord are lacking. Here, by injecting the cholera toxin B subunit into the sciatic nerve of a rhesus monkey, we successfully labeled the motor neurons and primary sensory afferents in the lumbar and sacralspinal cord. Labeled alpha motor neurons were located in lamina IX of the L6–S1 segments, which innervate both flexors and extensors. The labeled primary sensory afferents were mainly myelinated Aβ fibers that terminated mostly in laminae I and II of the L4–L7 segments. Together with the labeled proprioceptive afferents, the primary sensory afferents formed excitatory synapses with multiple types of spinal neurons. In summary, our methods successfully traced neuronal connections in the monkey spinal cord and can be used in spinal cord studies when nonhuman primates are used.

## Graphical abstract

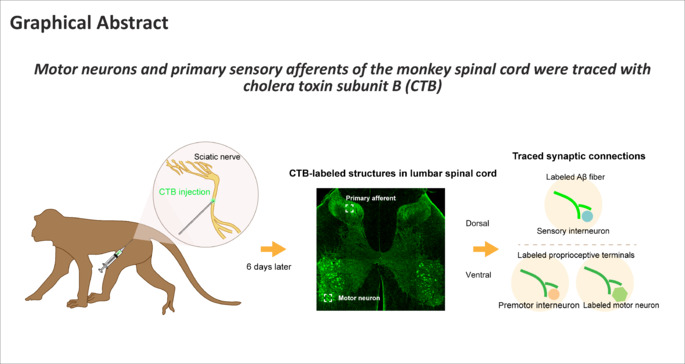

## Introduction

Nonhuman primates (NHPs) are more similar in neuroanatomy and neurophysiology to humans than to rodents (Capitanio and Emborg, 2008; Park and Silva, 2019; Feng et al., 2020; Neziri et al., 2024), and thus are being increasingly used as animal models in neuroscientific research. Researchers, clinicians and spinal cord injury (SCI) patients agreed that the safety and efficacy of cell transplantation should first be verified in SCI models generated from NHPs (Courtine et al., 2007; Kwon et al., 2012, 2013, 2015; Poulen and Perrin, 2024; Olaya et al., 2025). Neural progenitor cells, which can generate neurons to reconstruct damaged neural circuits, hold great promise for treating SCI (Fischer et al., 2020; Zipser et al., 2022; Ribeiro et al., 2023; Hosseini et al., 2024). For example, transplanted human neural stem cells derived from fetal spinal cord improved limb motor function in a rhesus monkey spinal cord hemisection model (Dulin et al., 2018; Rosenzweig et al., 2018), possibly through circuit reconstruction. The commonly used method for tracing neurons in SCI monkeys involves the injection of biotinylated dextran amine into the primary motor cortex to label the corticospinal tract to probe its regeneration and connection with grafted human neurons (Rosenzweig et al., 2018). However, the connections between human neurons and monkey motor neurons innervating specific muscles are unexplored. Furthermore, regeneration of ascending sensory axons that transmit tactile information and proprioception into grafts is important because tactile information and proprioception are integrated to achieve dynamic motor function. We and others have demonstrated that grafted human γ-aminobutyric acid (GABA) neurons alleviate neuropathic pain (Fandel et al., 2016) and spasm (Gong et al., 2021; Zheng et al., 2023a) after SCI. When using NHP models (Zheng et al., 2023b), methods are needed to show the connections between specific monkey motor neurons and primary sensory afferents with grafted human GABA neurons. These connections could be observed through tracing the motor neurons and primary sensory afferents of the monkey spinal cord; however, no such methods exist.

The cholera toxin B subunit (CTB) is a nontoxic component of the cholera toxin released by Vibrio cholerae, a gram-negative bacterium that causes profuse secretory diarrhea. CTB consists of five identical polypeptide chains noncovalently associated with a ring-shaped pentameric structure that mediates the binding of cholera toxin to the monosialoganglioside GM1 receptor, which is expressed on nerve fibers. Thus, CTB is widely used in neuroscience research as a retrograde and trans-ganglion tracer. In rats, intrasciatic nerve injection of CTB efficiently labels motor neurons and primary sensory afferents in the spinal cord (Shehab and Hughes, 2011; Hoeber et al., 2015; Lai et al., 2015). However, it is unknown whether this approach can be adapted for monkeys because of differences in the sizes of the nerve and spinal cord and possibly in the velocity of axonal transport. Here, we injected CTB into the bilateral sciatic nerves of one rhesus monkey to assess the labeling efficiency of CTB in NHPs and to further explore the local circuits involving the labeled structures.

## Methods

### Animals

One adult (11 years old, body weight 8.9 kg, stock No. T201301) male rhesus macaque (*Macaca mulatta*) was purchased from Hubei TopGene Biotechnology Co., Ltd. (Hubei, China, license No. SCXK (E) 2016-0010) and was the subject of this study. After arrival, the monkey was acclimated to the animal facility for at least 6 months before the experiments were initiated. The monkey was housed individually in a stainless-steel cage (70 cm high, 87 cm wide, and 61 cm deep) in a housing room with the temperature maintained at 22–24°C, a relative humidity of 45%–60%, and a 12-hour day/night cycle (lights on at 6:00 and off at 18:00). A metal mirror (15 cm diameter) was attached to the outside of the cage to allow the monkey to view activity in most regions of the room. A standard diet and drinking water were provided daily, and fruits and vegetables were added to supply vitamin C. In addition, fruits and peanuts were added to a hollow rubber KONG® toy to reward the monkey. All animal experiments were performed in accordance with the National Institutes of Health Guide for the Care and Use of Laboratory Animals (8^th^ ed., National Research Council, 2011), and were approved by the Committee on the Ethics of Animal Experiments and the Institutional Animal Care and Use Committee at Tongji Hospital, Tongji Medical College, Huazhong University of Science and Technology (approval number TJH-201903007) on March 1, 2019.

### Sciatic nerve injection of cholera toxin B subunit

**Figure 1:**
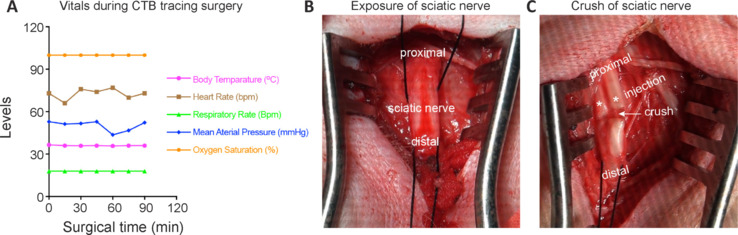
Injection of CTB into the monkey sciatic nerve. (A) Body temperature, heart rate, respiratory rate, mean arterial pressure and blood oxygen saturation, were recorded during surgery and CTB injection into monkey sciatic nerves. (B) Image showing the exposure of the monkey sciatic nerve. (C) Image showing the crush of the sciatic nerve before CTB injection. The white arrow indicates the crush site. The asterisks indicate the site of CTB injection. CTB: Cholera toxin subunit B.

After fasting for 12 hours to avoid aspiration complications, the monkey was anesthetized by intramuscular injection of tiletamine-zolazepam hydrochloride (0.5 mg/kg; Zoletil 50, Virbac, Carros, France), xylazine hydrochloride (0.5 mg/kg; Sigma-Aldrich, St. Louis, MO, USA, Cat# X1251), and atropine (0.05 mg/kg), and was then transferred from the home cage to the operating room and placed in the prone position. Body temperature was maintained with a heating pad. The fur on the haunch and forearm (to install an intravenous catheter) was shaved. Under aseptic conditions, a 20 G catheter was placed in the subcutaneous vein, and lactated Ringer’s solution (10 mL/kg/h) was given. Ceftriaxone (50 mg/kg) was administered for prophylaxis against infection. Vital signs, specifically body temperature, heart rate, respiratory rate, blood pressure and oxygen saturation, were closely monitored and maintained within an accepted range (**Figure 1A**). After intravenous infusion of propofol (5 mg/kg), the monkey was intubated and maintained at the surgical plane of anesthesia with 1.5%–2% isoflurane (RWD, Shenzhen, China) and a constant rate intravenous infusion of fentanyl citrate (0.01 mg/kg/h, Yichang Humanwell Pharmaceutical Co., Ltd., Hubei, China).

A longitudinal incision was made in the skin over the base of the tail to the posterior superior iliac spine using a #21 surgical blade. The superficial fascia and gluteus maximus were bluntly separated with a vessel clamp and retracted with a retractor along the direction of the fibers to expose the sciatic nerve (**Figure 1B**). To minimize animal suffering, 2% lidocaine hydrochloride was applied to the sciatic nerve with a cotton swab. The sciatic nerve was crushed with a 2-mm wide blunt vessel clamp for 60 seconds to facilitate maximal contact between the tracer and the nerve fibers (**Figure 1C**). Complete crushing was confirmed by the presence of a translucent band across the nerve. A 100-μL microsyringe (Cat# 7656-01, Hamilton, Reno, NV, USA) was filled with 0.1% CTB (100 μL for the left side and 50 μL for the right side) (Cat# NBP2-61449, Novus Biologicals, Littleton, CO, USA). Through a removable needle compression fitting (Cat# 55750-01, Hamilton), the microsyringe was connected with a beveled glass micropipette with a tip diameter of 40 μm. The micropipette was made from a glass capillary (Cat# 1B100F-4, World Precision Instruments, Sarasota, FL, USA) using a Flaming/Brown Micropipette Puller (PC-97, Sutter Instruments, Novato, CA, USA) and a Micro Grinder (Cat# EG-402, Narishige, Tokyo, Japan). Under a surgical microscope (PSMB5N, World Precision Instruments), the needle tip of the micropipette was inserted into the nerve just proximal to the crush site. CTB was slowly injected over approximately 2 minutes. The needle tip was left in place for 3 to 4 minutes and was removed slowly over 15 to 20 seconds to prevent leakage of the injection solution. Following injection, the nerve was ligated between the site of inoculation and the distal part of the nerve. The superficial fascia and gluteus maximus were sutured with 3-0 interrupted sutures. The skin was sutured with 2-0 interrupted sutures. Using the same method, CTB was also injected into the sciatic nerve on the contralateral side. The monkey was extubated, allowed to recover, and subsequently returned to the home cage. Ceftriaxone (50 mg/kg) was intramuscularly administered daily for 3 days. Meloxicam (5 mg/kg) was subcutaneously administered daily for 3 days. The incision site was sterilized daily with iodophor for 3 days.

### Tissue processing

Six days after sciatic nerve injection of CTB, the monkey was sedated by intramuscular injection of tiletamine-zolazepam (0.5 mg/kg; Zoletil 50, Virbac), xylazine hydrochloride (0.5 mg/kg; Sigma-Aldrich, Cat# X1251) and atropine (0.05 mg/kg), euthanized by intravenous infusion of sodium pentobarbital (60 mg/kg; Fujian Mindong Rejuvenation Pharmaceutical CO., LTD, Fujian, China), and perfused through the heart with 4 L of heparinized (100,000 U/L) normal saline, followed by 4 L of 4% paraformaldehyde in phosphate-buffered saline. The entire brain and spinal cord were harvested and postfixed in the same fixative at 4°C for 24 hours and then dehydrated in 4 L of 30% sucrose solution at 4°C for 72 hours. The thoracic (T)12 to sacral (S)1 segments of the spinal cord were dissected, embedded in optimal cutting temperature compound (Sakura Finetek, Torrance, CA, USA, Cat# 4853), and flash frozen. A cryostat microtome (CM1950, Leica, Nussloch, Germany) was used to cut serial coronal sections, 30-μm thick, which were collected into cryoprotectant consisting of 30% sucrose and 30% ethylene glycol in phosphate-buffered saline and stored at –80°C.

### Immunofluorescence

Free-floating sections were washed three times with Tris-buffered saline containing 0.25% Triton-X-100 (TBST) for 5 minutes each, blocked with QuickBlock^TM^ blocking buffer (Cat# P0260, Beyotime, Zhejiang, China) for 15 minutes, and incubated overnight (for more than 16 hours) with primary antibodies (**Table 1**) in QuickBlock^TM^ primary antibody dilution buffer (Cat# P0262, Beyotime) at 4°C. Thereafter, the sections were rinsed three times with TBST for 5 minutes each and then incubated with fluorochrome-conjugated secondary antibodies (**Table 2**) and Hoechst 33258 (Sigma, Cat# 94403) in QuickBlock^TM^ secondary antibody dilution buffer (Beyotime, Cat# P0265) in the dark for 1 hour. Finally, the sections were washed three times with TBST for 5 minutes each, mounted onto slides, air-dried thoroughly, and cover-slipped with Prolong Glass Antifade Mount (Cat# P36980, Invitrogen, Carlsbad, CA, USA).

**Table 1:** Primary antibodies used in immunofluorescence analysis

	Species/dilution	Supplier	Cat#/RRID
SOX9	Goat/1:200	R&D Systems, Minneapolis, MN, USA	AF3075/AB_2194160
GFAP	Rabbit/1:1000	Cell Signaling Technology, Danvers, MA, USA	12389/AB_2631098
OLIG2	Goat/1:200	R&D Systems	AF2418/AB_2157554
SOX10	Goat/1:1000	R&D Systems	AF2864/AB_442208
IBA1	Goat/1:1000	Abcam, Cambridge, UK	ab5076/AB_2224402
P2RY12	Rabbit/1:1000	AnaSpec, Fremont, CA, USA	55043A/AB_2298886
NeuN	Chicken/1:1000	Millipore, Burlington, MA, USA	ABN91/AB_11205760
NeuN	Guinea pig/1:1000	Millipore	ABN90/AB_11205592
FOXP2	Rabbit/1:2000	Abcam	ab16046/AB_2107107
SATB1	Rabbit/1:50	Abcam	ab109122/AB_10862207
ISLET1	Rabbit/1:1000	Abcam	ab109517/AB_10866454
ChAT	Rabbit/1:1000	Abcam	ab178850/AB_2721842
Calretinin	Rabbit/1:1000	Swant, Marly, Switzerland	CR 7697/AB_2619710
Calbindin	Rabbit/1:1000	Swant	CB38/AB_10000340
LMX1B	Rabbit/1:500	Abcam	ab259926
LHX1	Rabbit/1:200	Thermo Fisher Scientific, Waltham, MA, USA	PA5-78394/AB_2736089
CTB	Goat/1:500	List Biological, Campbell, CA, USA	703/AB_10013220
CTB	Rabbit/1:500	Sigma-Aldrich, St. Louis, MO, USA	C3062/AB_258833
CGRP	Rabbit/1:2000	Peninsula Lab, San Carlos, CA, USA	T-4032/AB_518147
IB4-Biotin	1:500	Thermo Fisher Scientific	I21414
Galanin	Rabbit/1:1000	Abcam	ab254556/AB_2889640
NPY	Rabbit/1:2000	ImmunoStar, Hudson, WI, USA	22940/AB_10720817
Synaptophysin	Mouse/1:2000	Millipore	MAB329/AB_94786
VGLUT1	Guinea pig/1:1000	Millipore	AB5905/AB_2301751
VGLUT2	Rabbit/1:200	Abcam	ab216463/AB_2893024
VGLUT3	Guinea pig/1:200	Millipore	AB5421-I/AB_2819014
GAD65	Rabbit/1:1000	Abcam	ab239372
VAChT	Rabbit/1:1000	Synaptic Systems, Goettingen, Germany	139 103/AB_887864
Homer1	Rabbit/1:1000	Synaptic System	160 003/AB_887730
c-Caspase-3	Rabbit/1:500	Cell Signaling Technology	9661/AB_2341188

CGRP: Calcitonin gene-related peptide; ChAT: choline acetyltransferase; CTB: cholera toxin subunit B; FOXP2: forkhead box protein P2; GAD65: glutamic acid decarboxylase 65; GFAP: glial fibrillary acidic protein; IB4: isolectin B4; LHX1: LIM homeobox 1; LMX1B: LIM homeobox transcription factor 1 beta; NPY: neuropeptide Y; OLIG2: oligodendrocyte transcription factor 2; P2RY12: P2Y purinergic receptor 12; SATB1: special AT-rich sequence-binding protein 1; SOX9: SRY-box transcription factor 9; VAChT: vesicular acetylcholine transporter; VGLUT1: vesicular glutamate transporter 1; VGLUT2: vesicular glutamate transporter 2; VGLUT3: vesicular glutamate transporter 3.

**Table 2:** Secondary antibodies used in immunofluorescence analysis

	Dilution	Supplier	Cat#/RRID
Donkey anti-mouse Alexa Fluor 488	1:1000	Invitrogen, Carlsbad, CA, USA	A-21202/AB_141607
Donkey anti-mouse Alexa Fluor 647	1:1000	Invitrogen	A-31571/AB_162542
Donkey anti-rabbit Alexa Fluor 488	1:1000	Invitrogen	A-21206/AB_2535792
Donkey anti-rabbit Alexa Fluor 594	1:1000	Invitrogen	A-21207/AB_141637
Donkey anti-goat Alexa Fluor 488	1:1000	Invitrogen	A-11055/AB_2534102
Donkey anti-goat Alexa Fluor 594	1:1000	Invitrogen	A-11058/AB_2534105
Donkey anti-guinea pig Alexa Fluor 488	1:1000	Jackson ImmunoResearch, West Grove, PA, USA	706-545-148/AB_2340472
Donkey anti-guinea pig Cy3	1:1000	Jackson ImmunoResearch	706-165-148/AB_2340460
Donkey anti-guinea pig Alexa Fluor 647	1:1000	Jackson ImmunoResearch	706-605-148/AB_2340476
Donkey anti-chicken Cy3	1:1000	Jackson ImmunoResearch	703-165-155/AB_2340363

### Laser scanning confocal microscopy

Fluorescence images were acquired using a confocal microscope (Olympus, Tokyo, Japan, Fluor View FV3000). For the immunofluorescence staining of four markers, the fluorescent channels were grouped into two separate phases for sequential scanning: Hoechst 33258 and 594 were assigned to one phase, and 488 and 647 were assigned to another. In the case of five-marker immunofluorescence staining, the channels were divided into three phases: Hoechst 33258 and Cy3 were scanned together in the first phase, 488 and 594 in the second phase, and 647 was scanned separately in the third phase. Sequential scanning was used to avoid spectral overlap between the channels and ensure the clear separation of signals from each marker. The bandwidth of each channel was set as follows: Hoechst 33258, 430–470 nm; Alexa Fluor 488, 500–540 nm; Cy3, 570–580 nm; Alexa Fluor 594, 610–630 nm; and Alexa Fluor 647, 650–750 nm. The high voltage was set at 700, the gain was set at 1, and the offset was set at 10 for all channels. Under the Hi-Lo viewing mode, the laser intensity of each channel was adjusted to the maximum possible intensity to avoid photobleaching and overexposure. The scanning speed was set at 8 μs/pixel. To capture the entire coronal spinal cord section, multiple area time lapse imaging was used. The matrix was set to 5 × 5 areas in rows and columns, and each area was acquired under a 10× objective lens with an optical zoom set at 1 and a resolution set at 256 × 256 pixels. For the region of interest, z stacks of images were captured under a 20× or 100× objective lens, with the optical zoom set at 1–5 as needed and the resolution set at 1024 × 1024. The images were postprocessed and analyzed with Fiji (National Institutes of Health, Bethesda, MD, USA) (Schindelin et al., 2012). A total of three sections were quantified and averaged for each spinal segment. The area of the dorsal and ventral horns was delineated as laminae I to VI and laminae VII to IX, respectively. For quantifying the mean immunofluorescence intensity, the dorsal and ventral horns of L6 sections were analyzed and compared with randomly selected regions from the lesion core for each marker.

### Statistics analysis

No statistics were used to determine strategies for randomization, sample size estimation, or inclusion and exclusion of any data. No statistical analyses were performed. A bar graph was created using GraphPad Prism 8 (GraphPad Software, San Diego, CA, USA).

## Results

### Successful labeling of the monkey lumbosacral spinal cord via injection of cholera toxin B subunit into the sciatic nerve

**Figure 2:**
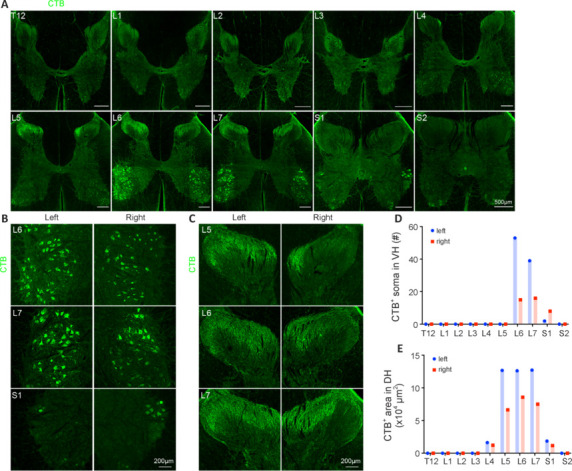
Successful labeling of the monkey lumbosacral spinal cord via injection of CTB into the sciatic nerve. (A) Representative images of monkey T12–S2 spinal cord coronal sections showing CTB-labeled cells in the ventral horn (VH) and structures in the dorsal horn (DH). (B) Magnified images of the ventral horn of the L6–S1 segments from A showing CTB-labeled cells. (C) Magnified images of the dorsal horn of segments L5–L7 from A showing CTB-labeled structures. (D) Quantification of the number of CTB-labeled cells in the ventral horn of the T12–S2 segments. (E) Quantification of the area of CTB-labeled structures in the dorsal horn of the T12–S2 segments. Scale bars: 500 μm in A, 200 μm in B and C. The CTB^+^ signals were represented in green. CTB: Cholera toxin subunit B.

We conducted the surgery and assessed the labeling efficiency 6 days later in one adult male rhesus monkey that was subjected to SCI followed by CTB injection. The surgery took less than 2 hours (**Figure 1A**). Body temperature, heart rate, respiratory rate, mean arterial pressure and blood oxygen saturation, were within the normal ranges (**Figure 1A**). After the sciatic nerve was exposed (**Figure 1B**), the sciatic nerve was crushed (**Figure 1C**), and CTB was subsequently injected into the proximal area of the crush site (**Figure 1C**). Six days later, the monkey was euthanized. The T12 to S2 segments of the spinal cord were serially cut into coronal sections and immunostained with an anti-CTB antibody. CTB^+^ signals could be clearly identified mainly in the lumbar (L)4–S1 spinal cord, in both the ventral and dorsal horns of gray matter but not in white matter (**Figure 2A**). In the ventral horn of the L6–S1 segments, CTB^+^ signals were more like cell somas, mainly in the L6 and L7 segments (**Figure 2B**). Quantification of the number of CTB-labeled cells confirmed that the L6 and L7 segments had the most CTB-labeled cells (**Figure 2D**). In the dorsal horn, CTB^+^ signals were densely intermingled, mainly in the L5–L7 segments and less so in the L4 and S1 segments (**Figure 2C**). Quantification of the area of CTB-labeled structures confirmed that the L5–L7 segments had the most CTB-labeled structures (**Figure 2E**). Taken together, these results suggested that the injection of CTB into the sciatic nerve labeled cells and structures of the lumbosacral spinal cord in a rhesus monkey.

### Cholera toxin B subunit–labeled cells in the ventral horn of the monkey lumbosacral spinal cord were motor neurons

**Figure 3:**
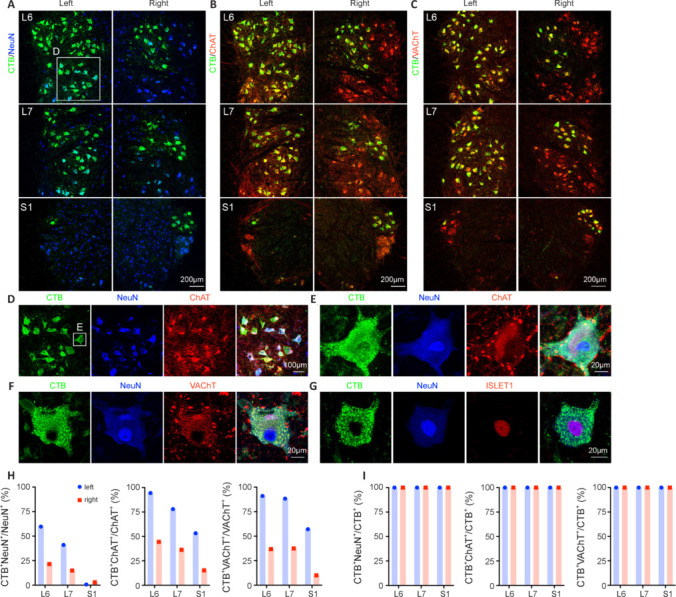
The CTB-labeled cells in the ventral horn of the monkey lumbosacral spinal cord were motor neurons. (A–C) Representative images of CTB and NeuN (A), ChAT (B), and VAChT (C) immunostaining in the ventral horn of the L6–S1 monkey spinal cord. (D) Magnified images from A showing CTB^+^ cells expressing NeuN and ChAT. (E) Magnified images from D showing CTB^+^ cells expressing NeuN and ChAT. (F) Representative images of CTB, NeuN and VAChT immunostaining showing CTB^+^ cells expressing VAChT. (G) Representative images of CTB, NeuN, and ISLET1 immunostaining showing that CTB^+^ cells expressed ISLET1, the transcription factor of motor neurons. (H) Quantification of the percentages of CTB^+^NeuN^+^, CTB^+^ChAT^+^ and CTB^+^VAChT^+^ cells among the NeuN^+^, ChAT^+^ and VAChT^+^ cells, respectively. (I) Quantification of the percentages of CTB^+^NeuN^+^, CTB^+^ChAT^+^ and CTB^+^VAChT^+^ cells among the CTB^+^ cells. Scale bars: 200 μm in A–C, 100 μm in D, and 20 μm in E–G. ChAT: Choline acetyltransferase; CTB: cholera toxin subunit B; NeuN: neuron-specific nuclear protein; VAChT: vesicular acetylcholine transporter. ISLET1: insulin gene enhanced protein 1.

To identify the CTB-labeled cells in the ventral horn, we first costained CTB with neuron-specific nuclear protein (NeuN), a neuronal marker. In the ventral horns of the L6–S1 segments, CTB-labeled somas were positive for NeuN (**Figure 3A** and **D**), suggesting that the CTB-labeled cells were neurons. Moreover, CTB-labeled neurons in the ventral horn of the L6–S1 segments were positive for choline acetyltransferase (ChAT) (**Figure 3B**, **D**, and **E**) and vesicular acetylcholine transporter (VAChT) (**Figure 3C** and **F**), indicating that the CTB-labeled cells were cholinergic neurons. CTB^+^ neurons were also positive for insulin gene enhanced protein 1 (ISLET1) (**Figure 3G**), a transcription factor of motor neurons. Quantification of the percentages of CTB^+^NeuN^+^, CTB^+^ChAT^+^ and CTB^+^VAChT^+^ cells among the NeuN^+^, ChAT^+^ and VAChT^+^ cells (**Figure 3H**) indicated that intrasciatic nerve injection of CTB did not label all of the neurons, or even motor neurons, in the ventral horn. However, quantification of the percentages of CTB^+^NeuN^+^, CTB^+^ChAT^+^ and CTB^+^VAChT^+^ cells among the CTB^+^ cells (**Figure 3I**) indicated that the CTB-labeled cells in the ventral horn were exclusively motor neurons.

**Figure 4:**
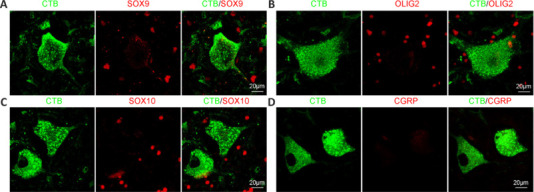
CTB-labeled cells in the monkey spinal cord were not glial cells. (A) Representative images showing that CTB^+^ cells were not positive for SOX9, a transcription factor of astrocytes. (B) Representative images showing that CTB^+^ cells did not express OLIG2, a marker of oligodendroglia lineage cells. (C) Representative images showing that CTB^+^ cells did not express SOX10, a marker of mature oligodendrocytes. (D) Representative images showing that CTB^+^ cells were negative for CGRP. Scale bars: 20 μm in A–D. CGRP: Calcitonin gene-related peptide; CTB: cholera toxin subunit B; OLIG2: oligodendrocyte transcription factor 2; SOX9: SRY-box transcription factor 9.

CTB-labeled somas were negative for astrocyte marker SOX9 (**Figure 4A**) and oligodendrocyte markers OLIG2 (**Figure 4B**) and SOX10 (**Figure 4C**), suggesting that no astrocytes or oligodendroglia were labeled with CTB. Although CTB-labeled rat spinal cord motor neurons have been reported to express calcitonin gene-related peptide (CGRP) (Cui et al., 2022), we did not observe CGRP expression in CTB-labeled monkey motor neurons (**Figure 4D**). Taken together, these results indicated that CTB injection into the sciatic nerve labeled motor neurons in the lumbosacral spinal cord in monkeys.

### Cholera toxin B subunit-labeled structures in the dorsal horn of the monkey lumbosacral spinal cord were primary sensory afferents

**Figure 5:**
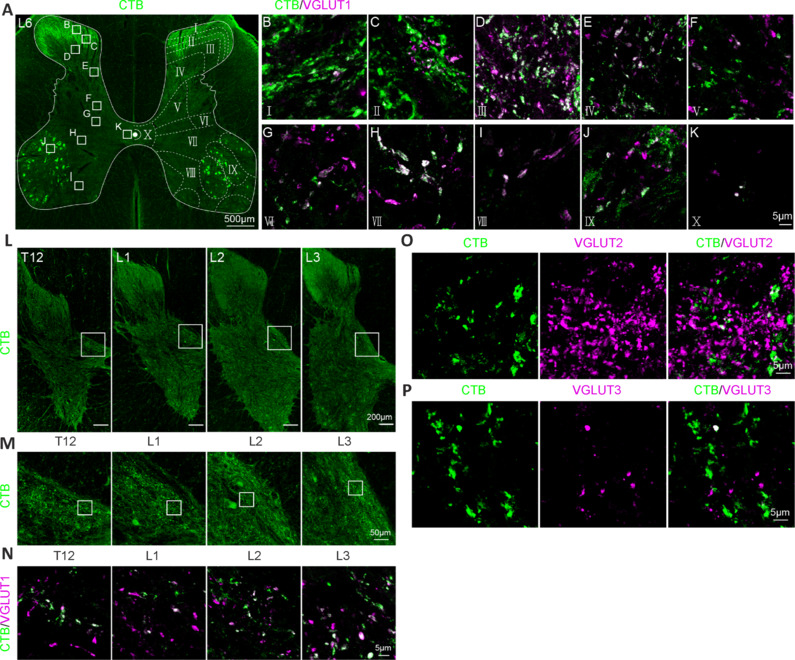
The CTB-labeled structures in the dorsal horn of the monkey lumbosacral spinal cord were primary sensory afferents. (A) Representative image of CTB immunostaining of the monkey L6 spinal cord. (B–K) Magnified images from A showing that CTB^+^ terminals were present in laminae I–X and expressed VGLUT1. (L) Images of CTB immunostaining in monkey T12–L3 spinal cord gray matter. (M) Magnified images from A showing that some CTB^+^ terminals were present in the inner part of the intermediate gray matter. (N) Magnified images from B showing CTB^+^ terminals expressing VGLUT1. (O) CTB^+^ terminals did not express VGLUT2. (P) A few CTB^+^ terminals expressed VGLUT3. Scale bars: 500 μm in A, 200 μm in L, 50 μm in M and 5 μm in B–K and N–P. CTB: cholera toxin subunit B; VGLUT: vesicular glutamate transporter.

We next determined the identity of CTB-labeled structures in the dorsal horn. Although CTB-labeled structures were mostly located in the dorsal horn (**Figure 5A**), at higher magnification, CTB-labeled axon terminals and punctate-like structures were also observed in all laminae of gray matter, including laminae I to X (**Figure 5B–K**). Moreover, these CTB^+^ terminals expressed vesicular glutamate transporter 1 (VGLUT1) (**Figure 5B–K**). Although CTB^+^ terminals were mostly present in the L4–S1 segments (**Figure 2E**), higher magnification of the inner part of the intermediate gray matter of the T12–L3 segments (**Figure 5L**) showed some CTB^+^ terminals (**Figure 5M**) that expressed VGLUT1 (**Figure 5N**). CTB^+^ terminals did not express VGLUT2 (**Figure 5O**), and a few CTB+ terminals expressed VGLUT3 (**Figure 5P**).

**Figure 6:**
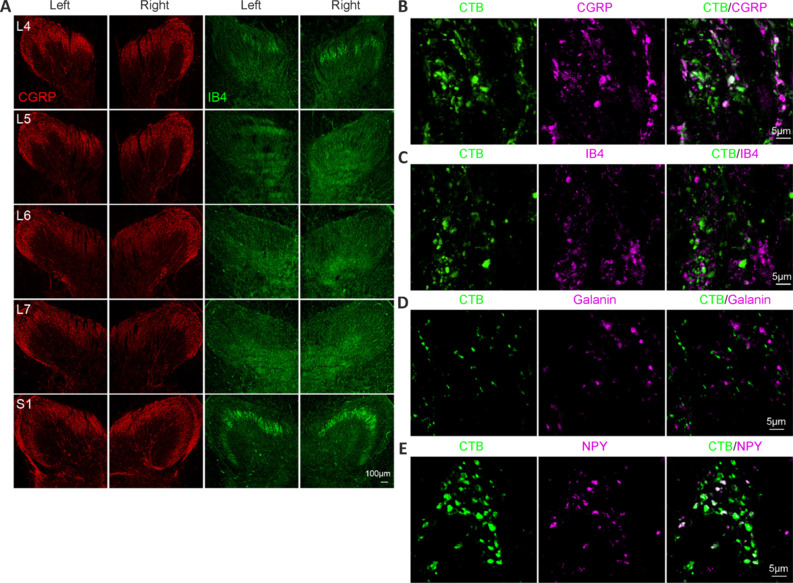
Presence of CTB-labeled primary sensory afferents in the monkey T12–L3 spinal cord. (A) Images of CGRP and IB4 immunostaining in the dorsal horn of the L4–S1 monkey spinal cord. CGRP^+^ fibers were present in all segments. IB4^+^ fibers were present in only the L4 and S1 segments and were not observed in the L5–L7 segments. (B) A few CTB^+^ terminals expressed CGRP. (C) No CTB^+^ terminals were positive for IB4. (D) CTB^+^ terminals did not express galanin. (E) CTB^+^ terminals exhibited sparse co-staining with NPY. Scale bars: 100 μm in A and 5 μm in B–E. CGRP: Calcitonin gene-related peptide; CTB: cholera toxin subunit B; IB4: isolectin B4; NPY: neuropeptide Y.

We next investigated whether CTB could label other primary sensory afferents. Many CGRP^+^ nonmyelinated peptidergic C fibers were present in the dorsal horn of the L4–S1 segments (**Figure 6A**), and a few of these fibers were labeled by CTB (**Figure 6B**). Isolectin B4 (IB4)^+^ nonmyelinated nonpeptidergic C fibers were present only in the L4 and S1 segments and were not observed in the L5–L7 segments (**Figure 6A**). In the L4 segments, IB4^+^ fibers were not labeled by CTB (**Figure 6C**). Although there are reports of CTB labeling in galanin^+^ and neuropeptide Y (NPY)^+^ peptidergic fibers in the rat spinal cord (Bao et al., 2002; Shehab et al., 2003), we observed that no monkey galanin^+^ (**Figure 6D**) and few NPY^+^ (**Figure 6E**) fibers were labeled with CTB. Taken together, these results suggested that CTB injection into the sciatic nerve labeled myelinated primary sensory afferents in the lumbosacral spinal cord in monkeys.

### Cholera toxin B subunit-labeled primary sensory afferents formed synapses with multiple types of monkey spinal neurons

**Figure 7:**
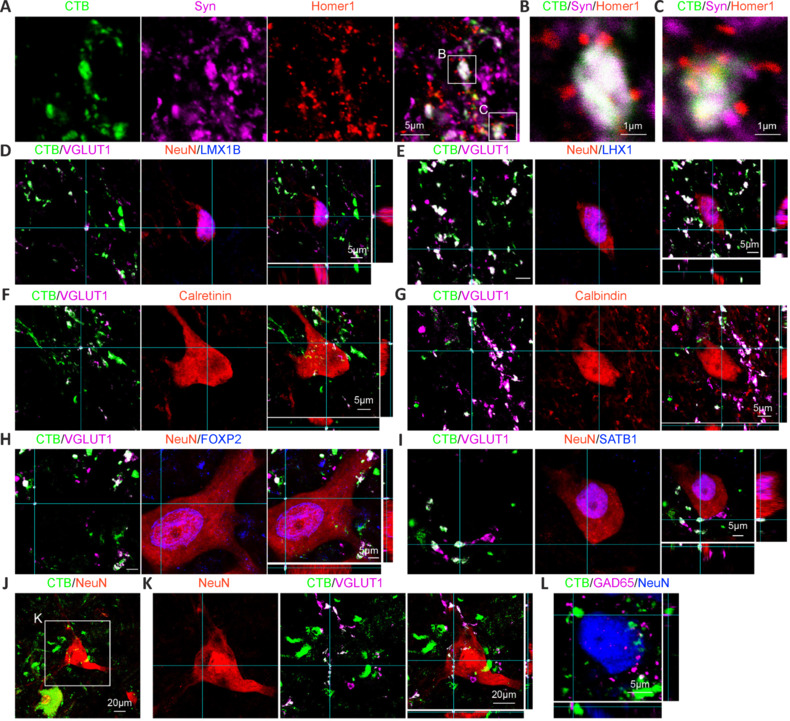
CTB-labeled primary sensory afferents form synapses with multiple types of monkey spinal neurons. (A) Representative images of CTB, Syn and Homer1 immunostaining showing that CTB^+^ terminals expressed the presynaptic marker Syn and were in close proximity to the postsynaptic marker Homer1. (B, C) Magnified images from A confirming that the CTB^+^ terminus expressed synaptophysin and was in close proximity to Homer1. (D–I) CTB^+^VGLUT1^+^ terminals formed synapses with LMX1B^+^ (D), LHX1^+^ (E), calretinin^+^ (F), calbindin^+^ (G), FOXP2^+^ (H) and SATB1^+^ (I) neurons. (J) Image of the ventral horn showing two motor neurons, one labeled with CTB and the other not labeled with CTB. (K) Magnified images from J showing that CTB^+^VGLUT1^+^ terminals formed synapses with motor neurons. (L) The CTB^+^ terminus was innervated by GAD65^+^ inhibitory synapses. Scale bars: 20 μm in J and K; 5 μm in A, D–I, and L; and 1 μm in B and C. CTB: Cholera toxin subunit B; FOXP2: forkhead box protein P2; GAD65: glutamic acid decarboxylase 65; LHX1: LIM homeobox 1; LMX1B: LIM homeobox transcription factor 1 beta; SATB1: special AT-rich sequence-binding protein 1; Syn: synaptophysin; VGLUT1: vesicular glutamate transporter 1.

Primary sensory afferents form synapses with multiple sensory and motor-related neurons in the rodent spinal cord. Thus, we examined the postsynaptic targets of monkey primary sensory afferents labeled with CTB. Monkey CTB^+^ terminals expressed the presynaptic marker synaptophysin (Syn) (**Figure 7A**) and were in close proximity to the postsynaptic marker Homer1 (**Figure 7B** and **C**), suggesting that CTB^+^ terminals indeed formed synapses. Then, we checked the postsynaptic neurons of monkey primary sensory afferents along the dorsal ventral axis. Monkey CTB^+^ terminals formed synapses with both LMX1B^+^ excitatory (**Figure 7D**) and LHX1^+^ inhibitory (**Figure 7E**) interneurons. In the dorsal gray matter, CTB^+^ terminals, including calretinin (CR)^+^ (**Figure 7F**) and calbindin (CB)^+^ (**Figure 7G**) neurons, formed synapses with sensory-associated interneurons. In the ventral gray matter, CTB^+^ terminals formed synapses with motor-related interneurons, specifically FOXP2^+^ (**Figure 7H**) and SATB1^+^ (**Figure 7I**) neurons. Moreover, in the motor neuron pool, CTB^+^ terminals appeared to contact motor neurons (**Figure 7J**) and form synapses with motor neurons (**Figure 7K**). Finally, monkey CTB^+^ terminals were innervated by glutamate decarboxylase 65 (GAD65)^+^ inhibitory synapses (**Figure 7L**). Taken together, these results suggested that CTB-labeled monkey primary sensory afferents formed synapses with multiple types of spinal neurons.

### Injection of cholera toxin B subunit into the sciatic nerve did not cause glial activation or apoptosis of motor neurons in the monkey spinal cord

**Figure 8:**
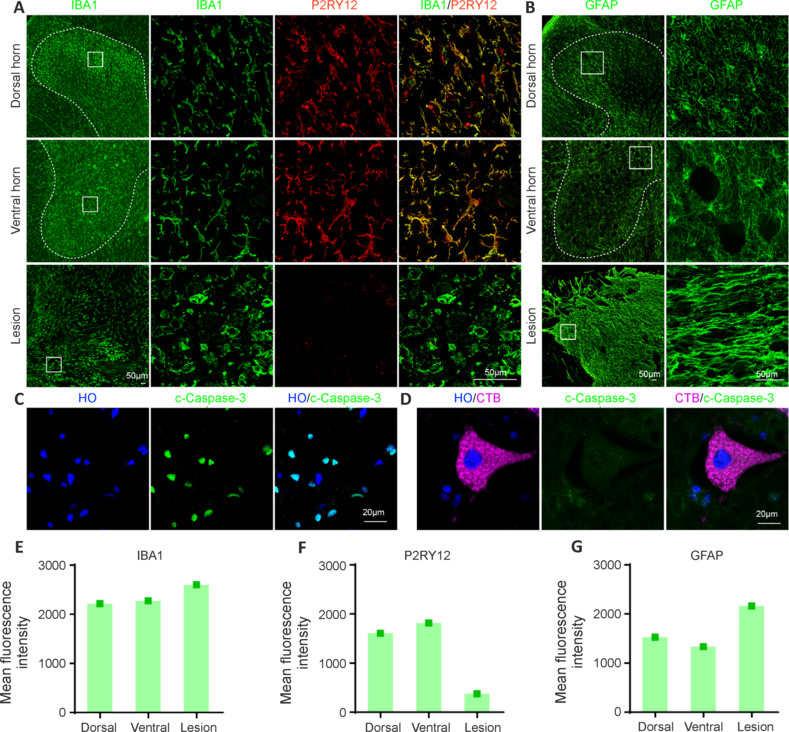
Injection of CTB into the sciatic nerve did not cause glial activation or apoptosis of motor neurons in the monkey spinal cord. (A) Representative images of IBA1 and P2RY12 immunostaining in the dorsal and ventral horns where CTB was labeled and in the spinal lesion site. (B) Representative images of GFAP immunostaining in the dorsal and ventral horns where CTB was labeled and in the spinal lesion site. The dotted lines in A and B delineate the spinal gray matter. (C) Representative images of c-Caspase-3 immunostaining showing that the cells were undergoing apoptosis. (D) Representative images of c-Caspase-3 and CTB immunostaining showing that CTB^+^ cells were not undergoing apoptosis. (E–G) Quantification of mean fluorescence intensity of IBA1 (E), P2RY12 (F) and GFAP (G) in the lumbar spinal dorsal horn (dorsal), ventral horn (ventral) and spinal lesion site (lesion). Scale bars: 50 μm in A and B, 20 μm in C and D. CTB: Cholera toxin subunit B; GFAP: glial fibrillary acidic protein; IBA1: ionized calcium binding adaptor molecule 1; P2RY12: P2Y purinergic receptor 12. HO: hoechst.

Because CTB is a protein produced by cholera toxin that can cause inflammation and lead to cell death, we investigated the safety of injecting CTB into monkey sciatic nerves. In both the dorsal and ventral horns where CTB-labeled primary sensory afferents and motor neurons were present, ionized calcium binding adaptor molecule 1 (IBA1)^+^ microglia had small cell somas and thin ramified processes, with evident expression of the homeostatic marker P2RY12 (**Figure 8A**). However, around the SCI lesion site, IBA1^+^ microglia were round and had larger cell bodies without processes, and the expression of P2RY12 was almost completely absent (**Figure 8A**). Quantification of fluorescence intensity showed an increasing trend in IBA1 expression and a decreasing trend in P2RY12 in the spinal lesion site, compared to the dorsal and ventral horns with dense CTB signals (**Figure 8E** and **F**). However, these findings are descriptive in nature and lack statistical analysis. Thus, microglia around CTB^+^ terminals and motor neurons appeared to be inactive. Similarly, in the dorsal and ventral horns where CTB^+^ terminals and motor neurons were present, glial fibrillary acidic protein (GFAP)^+^ astrocytes exhibited many thin and ramified processes (**Figure 8B**). However, around the SCI lesion site, the GFAP^+^ astrocytes had thick and parallel processes (**Figure 8B**), suggesting that they were activated and formed astroglial scars. Fluorescence analysis suggested a trend of elevated GFAP expression in the spinal lesion site, compared with the dorsal and ventral horns with dense CTB signals (**Figure 8G**). Thus, these findings suggest that astrocytes around CTB^+^ terminals and motor neurons were also quiescent. Finally, although cleaved caspase-3 (c-Caspas-3)^+^ cells that were undergoing apoptosis were present (**Figure 8C**), CTB-labeled motor neurons did not express c-Caspase-3 (**Figure 8D**), indicating that CTB^+^ motor neurons were not undergoing apoptosis. Taken together, these results suggested that CTB injection into the monkey sciatic nerve did not cause glial activation or apoptosis of motor neurons in the spinal cord.

## Discussion

Here, we showed that intrasciatic nerve injection of CTB labeled motor neurons and primary sensory afferents in the monkey lumbosacral spinal cord, consistent with the results in rats. To the best of our knowledge, only one study was published more than two decades ago (Tong et al., 1999), in which CTB was injected into the monkey sciatic nerve and the primary sensory afferents were labeled. However, the identity and distribution of these cells along the rostral-caudal segments and dorsal-ventral laminae of the spinal cord were unclear. Moreover, whether cells were labeled with CTB and, if so, their identity and distribution were also unknown. Thus, our study is the first to show the successful tracing of motor neurons and primary sensory afferents, as well as their distribution and connections, in the monkey lumbosacral spinal cord following CTB injection into the sciatic nerves.

In the previous study in which CTB was injected into monkey sciatic nerves (Tong et al., 1999), it was declared that many motor neurons were labeled with CTB, but no results were presented. Our study is the first to provide definite evidence that CTB injection into the sciatic nerves labels motor neurons in the lumbosacral spinal cord in monkeys. The CTB-labeled cells were present in the ventral horn, had large cell somas, and specifically expressed NeuN, ChAT, VAChT, and ISLET1, which confirmed their identity as motor neurons. The CTB-labeled motor neurons were likely alpha motor neurons because they had large cell bodies and all expressed NeuN, whereas gamma motor neurons have small cell bodies and do not express NeuN (Khan et al., 2022), although both alpha and gamma motor neurons express ChAT and VAChT. According to the atlas of the rhesus monkey spinal cord (Watson et al., 2021), our CTB-labeled motor neurons in the L6–S1 segments were those innervating the gluteal muscle, crural extensors and flexors, hamstrings, sartorius, gracilis and semitendinosus muscles, but not the axial muscles. In a previous anatomical study (Capogrosso et al., 2016), retrograde tracing via Fast Blue or Fluoro-Ruby injection into selected hindlimb muscles of rhesus monkeys showed that motor neurons innervating the gastrocnemius medialis and semitendinosus, but not the gluteus medius, iliopsoas, rectus femoris, tibialis anterior, extensor digitorum longus, or flexor hallucis longus, were labeled in the L6–S1 segments. Functionally, intraspinal microstimulation of gray matter in the L6–S1 spinal cord segments of rhesus monkeys evoked muscle activity in the semimembranosus, sartorius, vastus lateralis and medialis, biceps femoris, gastrocnemius, and tibialis anterior muscles, leading to hip extension, knee flexion, ankle extension and toe flexion and extension (Toossi et al., 2019). Thus, intrasciatic nerve injection of CTB labeled many alpha motor neurons in the L6–S1 spinal cord, which, when combined with the atlas of the rhesus monkey spinal cord and other anatomical and functional results, will be useful for targeting specific populations of motor neurons that innervate specific muscles of interest.

In the previous study in which CTB was injected into monkey sciatic nerves (Tong et al., 1999), fibers in the dorsal horn were labeled with CTB, but their distribution and identity were unclear. For the first time, we showed that many CTB-labeled fibers were present in the L4–S1 segments and were especially abundant in the L5–L7 segments. That study also reported moderate CTB labeling in laminae I and II, scattered CTB-positive nerve fibers in laminae III and IV, and no CTB labeling in laminae V and VI. However, we observed denser CTB labeling in laminae I and II than in laminae III and IV. Furthermore, scattered CTB labeling was present in laminae III to X. This could be explained by the fact that we crushed the nerve before injection, because the pioneering study (Tong et al., 1999) showed increased uptake and transport of CTB in dorsal root ganglion neurons after nerve transection. CTB-labeled fibers mainly expressed VGLUT1, with only a very small amount expressing VGLUT3 and CGRP, and none of them expressed VGLUT2 or IB4. In rodent spinal cord primary sensory afferents, VGLUT1 is expressed in touch-sensing fibers (heavily myelinated Aβ fibers with low activation thresholds), whereas VGLUT2 is expressed in thin, lightly myelinated Aδ fibers or unmyelinated C fibers that sense temperature and pain (Lagerström et al., 2010; Liu et al., 2010). VGLUT3 is expressed in a specific subpopulation of small sensory neurons that have unmyelinated C fibers termed low-threshold mechanoreceptors (C-LTMRs) (Larsson and Broman, 2019), which, unlike other C fibers, can transport CTB (Li et al., 2011). Thus, our CTB-labeled monkey fibers could mostly be Aβ fibers, with a few C-LTMRs, but not Aδ fibers or C fibers. In addition to cutaneous afferents, scattered CTB-labeled terminals were also present in the inner part of the intermediate gray matter of the T12–L3 segments, which was consistent with previous results in rats (Shehab and Hughes, 2011). These CTB-labeled terminals, like those present in the ventral laminae in the L4–S1 segments, could be proprioceptor afferents originating from muscle spindles (Shehab and Hughes, 2011).

As in rodents (Peirs et al., 2020), CTB^+^ monkey sensory terminals form excitatory synapses with multiple types of spinal neurons, including both LMX1B^+^ excitatory (Ding et al., 2004; Rayon et al., 2021; Yadav et al., 2023) and LHX1^+^ inhibitory (Pillai et al., 2007; Rayon et al., 2021; Yadav et al., 2023) interneurons. In the dorsal horn, CTB^+^ monkey cutaneous sensory terminals form synapses with CR^+^ neurons, which in rodents recruit spinoparabrachial neurons to elicit a nocifensive response (Petitjean et al., 2019; Smith et al., 2019). In the intermediate and ventral gray matter, CTB^+^ monkey proprioceptive terminals formed synapses with SATB1^+^ and FOXP2^+^ interneurons and motor neurons. SATB1^+^ neurons in the rodent spinal cord were identified as motor synergy encoder neurons, which represent central nodes in neural pathways for voluntary and reflexive movement (Levine et al., 2014). The circuit between CTB^+^ proprioceptive terminals and FOXP2^+^ V1 neurons could mediate the reciprocal inhibition of motor neurons that mediates flexor-extensor alternations (Bikoff et al., 2016; Falgairolle and O’Donovan, 2019), whereas the circuit between CTB^+^ proprioceptive terminals and motor neurons mediates the monosynaptic Hoffman reflex (Benson et al., 2024). In addition, monkey CTB^+^ terminals were innervated by GAD65^+^ inhibitory synapses, which could be similar to the presynaptic inhibition structure in rodents that originates from GABA neurons within either the spinal cord (Boyle et al., 2019) or the rostral ventromedial medulla (De Preter and Heinricher, 2024).

There are several methodological aspects to consider. First, CTB has been shown to label primary sensory afferents after injection into either uninjured or injured (transected or crushed) rats (Tong et al., 1999; Bao et al., 2002; Shehab et al., 2003; Shehab and Hughes, 2011; Hoeber et al., 2015; Lai et al., 2015) or monkeys (Tong et al., 1999). However, when CTB was injected into the injured sciatic nerve, its uptake by axons and transport in dorsal root ganglion neurons increased, and its labeling was greater in sciatic nerve-injured rats than in rats with uninjured sciatic nerves (Tong et al., 1999). Thus, in this study, the monkey sciatic nerves were crushed before the injection of CTB, as in rats (Lai et al., 2015), to maximize the uptake of CTB. Many galanin^+^ and NPY^+^ peptidergic fibers in laminae I and II were labeled by CTB when injected into transected sciatic nerves of rats (Bao et al., 2002; Shehab et al., 2003). However, in the present study, CTB injected into crushed monkey sciatic nerves labeled no galanin^+^ and few NPY^+^ fibers. The reason for these differences could be that we immediately injected CTB after sciatic nerve crush in monkeys, whereas in rats (Tong et al., 1999; Bao et al., 2002; Shehab et al., 2003), CTB was injected at least 14 days after transection when neuroplasticity had occurred. Indeed, CTB injected into rat sciatic nerves 2 days after transection (Bao et al., 2002) did not lead to increased labeling of fibers in laminae I and II. Another concern regarding CTB injection into crushed sciatic nerves is transneuronal tracing, which results in the labeling of GABA interneurons in all laminae of gray matter in the C7–S1 segments in rats (Lai et al., 2015). However, our CTB-labeled neurons were observed to be only alpha motor neurons that were present only in laminae IX of the L7–S1 segments, and showed no transneuronal tracing of other neurons in other laminae and segments. Differences in animal species or even types of CTB used might account for the disparity in the results. The CTB that we used was a recombinant protein without conjugates. Other types of recombinant CTB, such as biotin- or fluorochrome-conjugated recombinant CTB, used in rats (Conte et al., 2009a, b; Lai et al., 2015; Cui et al., 2022) could also be applied in monkeys. Finally, the concentration of CTB used in most of the previous studies was 1%, whereas in the present study, the CTB concentration was 0.1%. Nerve crush injury improved uptake to achieve dense CTB labeling, which could reduce the requisite amount of CTB.

The ethical issues involved in this study should be emphasized. With greater similarities to humans, NHPs are hence considered the ultimate animal model for various diseases, especially for neurological disorders (Courtine et al., 2007; Capitanio and Emborg, 2008; Kwon et al., 2012, 2013, 2015; Park and Silva, 2019; Feng et al., 2020; Neziri et al., 2024; Poulen and Perrin, 2024; Olaya et al., 2025). Our study addresses the technical gap in tracing primary afferents and motor neurons with CTB in NHPs, thereby facilitating essential circuit exploration in future studies involving these models. Despite the substantial scientific benefits, it is crucial to balance these advantages with a thorough consideration of animal welfare. In our study, all procedures involving the monkey were conducted in accordance with the ethical guidelines set forth by the approving committee, which included environment enrichment, regular veterinary care, surgical operation with minimal harm, and postoperative management. To minimize stress, we provided environmental enrichments, such as mirrors for observation and food-dispensing toys. The monkey received a balanced diet, supplemented with fresh fruits and vegetables rich in vitamin C. Under the guide of an experienced veterinarian, dedicated staff closely monitored the animal health and well-being. Moreover, the invasive procedures were conducted by a qualified neurosurgeon in a specialized surgical facility of large animals, strictly following sterile and minimally invasive principles. Postoperative care was provided with a focus on effective pain management, infection control, and nursing interventions to ensure animal comfort. Taken together, our study maintains a steadfast commitment to animal welfare and ethical integrity while contributing to scientific advancements.

The limitations of this study need to be addressed. The successful tracing of motor neurons and primary sensory afferents in the monkey spinal cord was demonstrated in only one animal, primarily because of the high value of the rhesus monkeys and study design in compliance with ethical guidelines. However, we believe that this method would be reproducible in additional animals. This will be fulfilled in our ongoing work where motor neurons and primary sensory afferents in the spinal cord of SCI monkeys will be traced via CTB to visualize their connections with transplanted human neurons. With regards to the present study, caution is warranted because of the potential bias from our single-sample experiment, especially when assessing the impact of sciatic nerve crush. We crushed the nerve immediately before CTB injection to enhance its uptake and transport, resulting in significantly denser labeling than was observed in the previous study with uninjured nerves (Tong et al., 1999). However, this procedure may also introduce variations due to neural plasticity. As reported in rodent studies, additional peptidergic afferents were labeled with CTB when injected at 2 weeks after nerve transection (Bao et al., 2002; Shehab et al., 2003). In our research, few peptidergic afferents were labeled by CTB, which we hypothesize is due to the significantly shorter time window between nerve crush and injection that was potentially insufficient for neural plasticity to occur. This negative result should be interpreted with caution until replicated in larger samples. Moreover, one rodent study using biotin-conjugated CTB reported extensive transneuronal tracing after sciatic nerve crush, possibly due to the enhanced neuronal expression of GM1 post-injury (Lai et al., 2015). Although we observed only labeled somas in lamina IX, indicating no sign of transneuronal tracing in the monkey, this negative finding also requires validation in more samples. We will continue to address these points in our ongoing work and look forward to engaging with other research teams using this tracing method.

In summary, we traced motor neurons and primary sensory afferents in the lumbosacral spinal cord via the injection of CTB into sciatic nerves. This method could be widely used in studies involving the monkey spinal cord, including SCI, amyotrophic lateral sclerosis and neuropathic pain studies, to reveal the projections and connections of targeted neurons and axons.

## Data Availability

*All the data reported in this paper will be shared by the lead contact upon request. This paper does not report the original code. Any additional information needed to reanalyze the data reported in this paper is available from the lead contact upon request*.
